# Tyrosine metabolic reprogramming coordinated with the tricarboxylic acid cycle to drive glioma immune evasion by regulating PD‐L1 expression

**DOI:** 10.1002/ibra.12107

**Published:** 2023-05-22

**Authors:** Ji‐Yan Wang, Xin‐Tong Dai, Qing‐Le Gao, Hong‐Kai Chang, Shuai Zhang, Chang‐Liang Shan, Tao He

**Affiliations:** ^1^ State Key Laboratory of Medicinal Chemical Biology, College of Pharmacy and Tianjin Key Laboratory of Molecular Drug Research Nankai University Tianjin China; ^2^ School of Integrative Medicine Tianjin University of Traditional Chinese Medicine Tianjin China; ^3^ Department of Pathology Characteristic Medical Center of The Chinese People's Armed Police Force Tianjin China

**Keywords:** fumarate, glioma, immune evasion, tyrosine metabolism, α‐KG

## Abstract

Due to the existence of the blood–brain barrier in glioma, traditional drug therapy has a poor therapeutic outcome. Emerging immunotherapy has been shown to have satisfactory therapeutic effects in solid tumors, and it is clinically instructive to explore the possibility of immunotherapy in glioma. We performed a retrospective analysis of RNA‐seq data and clinical information in 1027 glioma patients, utilizing machine learning to explore the relationship between tyrosine metabolizing enzymes and clinical characteristics. In addition, we also assessed the role of tyrosine metabolizing enzymes in the immune microenvironment including immune infiltration and immune evasion. Highly expressed tyrosine metabolizing enzymes 4‐hydroxyphenylpyruvate dioxygenase, homogentisate 1,2‐dioxygenase, and fumarylacetoacetate hydrolase not only promote the malignant phenotype of glioma but are also closely related to poor prognosis. The expression of tyrosine metabolizing enzymes could distinguish the malignancy degree of glioma. More importantly, tyrosine metabolizing enzymes regulate the adaptive immune process in glioma. Mechanistically, multiple metabolic enzymes remodel fumarate metabolism, promote α‐ketoglutarate production, induce programmed death‐ligand 1 expression, and help glioma evade immune surveillance. Our data suggest that the metabolic subclass driven by tyrosine metabolism provides promising targets for the immunotherapy of glioma.

## Graphical abstract

Due to the blood–brain barrier and high tumor heterogeneity, the clinical outcomes of glioma are worrisome. Extensive research work has identified several molecular subtypes and classifications, but these criteria have many limitations that make them ineffective in guiding treatment courses. We found that tyrosine metabolism was not only associated with a patient's malignant phenotype but also indicated survival prognosis. More importantly, tyrosine metabolism regulates the tumor microenvironment (TME) of glioma, promoting immune infiltration and immune evasion. In‐depth studies have found that the metabolic remodeling of tyrosine metabolite fumarate couples with the tricarboxylic acid cycle, promotes the accumulation of α‐ketoglutarate, and regulates the expression of programmed death‐ligand 1. This study showed that tyrosine metabolizing enzymes help glioma evade immune surveillance and increase their malignancy by regulating the TME, which can be developed into immunotherapy targets. Glioma is characterized based on the expression characteristics of tyrosine metabolizing enzymes, emphasizing a deep insight into its clinical characterization and TME to provide prognostic and therapeutic implications.

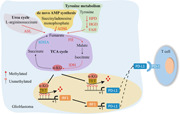

## 1 INTRODUCTION

Glioma is a common primary tumor in the brain, accounting for about 80% of brain tumors,^1^ which has a high incidence and mortality rate and is notorious for drug resistance and incurability.^2^ The main obstacle to the clinical treatment of glioma is tumor heterogeneity. Therefore, the clinical treatment of gliomas is largely dependent on molecular subtypes and classifications. Numerous studies on glioma focused on the subtypes and classifications. In 2016, the World Health Organization (WHO) identified malignant glioma as grades II, III, and IV based on histological features.^3^ It is common lore that the higher the grade, the higher the degree of tumor malignancy and the worse the patient's prognosis. However, grade classification does not fully reflect the heterogeneity of glioma, and this approach is limited by the subjectivity of neuropathologists. In addition to grade classification, molecular subtypes also have important guiding significance for the treatment of gliomas. The isocitrate dehydrogenase (IDH) mutation status has been found to be correlated with the prognosis of glioma patients,^4^, ^5^, ^6^ but IDH mutation occurs only in low‐grade gliomas (LGGs) and is not common in higher‐grade glioblastomas (GBM).^7^ The DNA repair gene O^6^‐methylguanine‐DNA methyltransferase (MGMT) is the most prominent epigenetically silenced gene in glioma.^8^, ^9^, ^10^ It has been reported that methylation of the MGMT promoter is an independent favorable prognostic factor for glioma and is beneficial to the clinical treatment of alkylating agent temozolomide (TMZ).^8^, ^11^ In addition, the codeletion status of chromosomes 1 and 19 (1p/19q) also has a good predictive value for the prognosis of glioma.^6^, ^12^, ^13^ This only occurs in IDH‐mutated gliomas.^14^ Although multiple molecular subtypes have been identified, the survival time of glioma patients has not improved significantly.^15^ Therefore, finding new classification criteria or novel biomarkers for glioma subtypes is an urgent need for clinical treatment.

Metabolic reprogramming is the alteration made by tumor cells in pursuit of rapid growth and proliferation. Glioma is no exception, and the brain itself is a highly metabolically active organ. It is well known that glucose is the main substrate as the energy source of brain cells. Besides, lactic acid, ketone bodies, fatty acids, and amino acids can also be used as fuel sources under certain circumstances.^16^, ^17^, ^18^ Arginine has been shown to modulate the tumor microenvironment (TME) of glioma and mediate immunotherapy.^19^ Fatty acid metabolism is also involved in the glioma TME and invasive processes.^20^ Therefore, looking for tumor biomarkers or new subtype classifications from the perspective of metabolism may be a hopeful direction.

In this study, based on the perspective of tyrosine metabolism, we found that the tyrosine metabolizing enzymes 4‐hydroxyphenylpyruvate dioxygenase (HPD), homogentisate 1,2‐dioxygenase (HGD), and fumarylacetoacetate hydrolase (FAH) were upregulated in more malignant glioma patients. Importantly, the expression of tyrosine metabolizing enzymes was able to discriminate the extent to which clinical features including grade, IDH status, 1p19q status, and MGMT, suggesting their potential as tumor markers in glioma. In addition, abnormal expression of tyrosine metabolizing enzymes (HPD, HGD, and FAH) alters the TME of glioma. Specifically, highly expressed tyrosine metabolizing enzymes not only promote immune infiltration but also induce the expression of immune checkpoints (programmed death‐ligand 1 [PD‐L1]), inhibit the activation of T cells, and increase the malignancy of tumors. In conclusion, we elucidated the expression characteristics of tyrosine metabolism in glioma, correlated with clinical features of patients, and participated in the immune infiltration and immune evasion, which will provide effective help for the immunotherapy of glioma.

## 2 MATERIALS AND METHODS

### 2.1 Data collection and collation

The gene expression data and clinical information of glioma patients in this study were downloaded from the Chinese Glioma Genome Atlas (CGGA) (http://www.cgga.org.cn/index.jsp) database. The CGGA database is a web application for data storage and analysis to explore brain tumor data sets over 2000 samples from Chinese cohorts.

### 2.2 Univariate Cox's regression analysis

In this study, we used SPSS (IBM SPSS Statistics for Windows, version 20.0; IBM Corp.) software to integrate survival time, survival status, and gene expression data, and assessed the prognostic significance of tyrosine metabolizing enzymes using univariate Cox's method. During the analysis, we removed patient samples with missing clinical information.

### 2.3 Patient grouping

We calculated the risk score of HPD, HGD, and FAH of the sample in the training set (CGGA, *n* = 273) and validation set (The Cancer Genome Atlas (TCGA), *n* = 562). The R package “survminer” was used to identify the optimal cutpoint of HPD, HGD, and FAH based on the expression level, the survival time, and the survival status. The expression level more than the optimal cutoff calculated by “survminer” was considered as the high‐expression group, but less than optimal cutoff was considered as the low‐expression group. Then, we plotted the survival curve to detect the correction between the high‐expression group and the low‐expression group. The survival prognosis of glioma patients was drawn by the R package “survival.”

### 2.4 Pathway enrichment analysis

Gene set enrichment analysis (GSEA) was performed using GSEA 4.0.3 (Broad Institute) in which the hallmark gene set “c5.go.v7.4.symbols.gmt” was adopted. We identified pathways with ∣normalized enrichment score∣ > 1 and *p* < 0.05 as potential candidate pathways.

### 2.5 Evaluation of immune infiltration

The R package “ESTIMATE” was designed to count scores for reflecting the infiltration levels of immune cells and stromal cells within the TME on the foundation of the specific gene expression level of immune and stromal cells.^21^ First, we used the “ESTIMATE” algorithm to calculate the tumor purity, ESTIMATE score, immune score, and stromal score of glioma patients in the training set (CGGA, *n* = 273) and the validation set (TCGA, *n* = 562). To investigate the difference in the infiltration level of immune cells, the R package “GSVA” (gene set variation analysis) was applied to count the proportion of 28 immune cells of all glioma samples on the foundation of the RNA‐seq file.^22^ According to the optimal cutpoint calculated by “survminer,” the above output data were divided into the high‐expression group and the low‐expression group. Then, the differences in immune scores and immune cell infiltration levels between the two groups were compared by using visual analysis tools.

### 2.6 GSVA

The expression level of immune checkpoint‐related genes was evaluated on the basis of the glioma sample expression file by the R package “GSVA.” The correlation between immune checkpoint‐related genes and HPD, HGD, and FAH was plotted by the R package “corrgram.”

### 2.7 Expression differences, mutation frequencies, and promoter methylation

The differential expression analysis of tyrosine metabolizing enzymes in LGG and low‐grade GBM was performed on the Gene Expression Profiling Interactive Analysis web server (http://gepia.cancer-pku.cn/index.html).^23^ The mutation of identified genes was identified and analyzed on the cBioPortal platform (https://www.cbioportal.org/).^24^, ^25^ The promoter methylation levels of IRF1 and CD274 were obtained from the CGGA database (http://www.cgga.org.cn/analyse/Methyl-data-distribution-result.jsp),^26^ respectively.

### 2.8 Statistical analysis

The diagram was drawn using GraphPad Prism 8 (GraphPad Software Inc.). All results were presented as mean ± standard deviation. A comparison of the two groups was made using two‐tailed Student's *t*‐test. A value of *p* < 0.05 was considered as the statistically significant threshold.

## 3 RESULTS

### 3.1 Tyrosine metabolism promotes a clinical malignant phenotype in glioma patients

**Figure 1:**
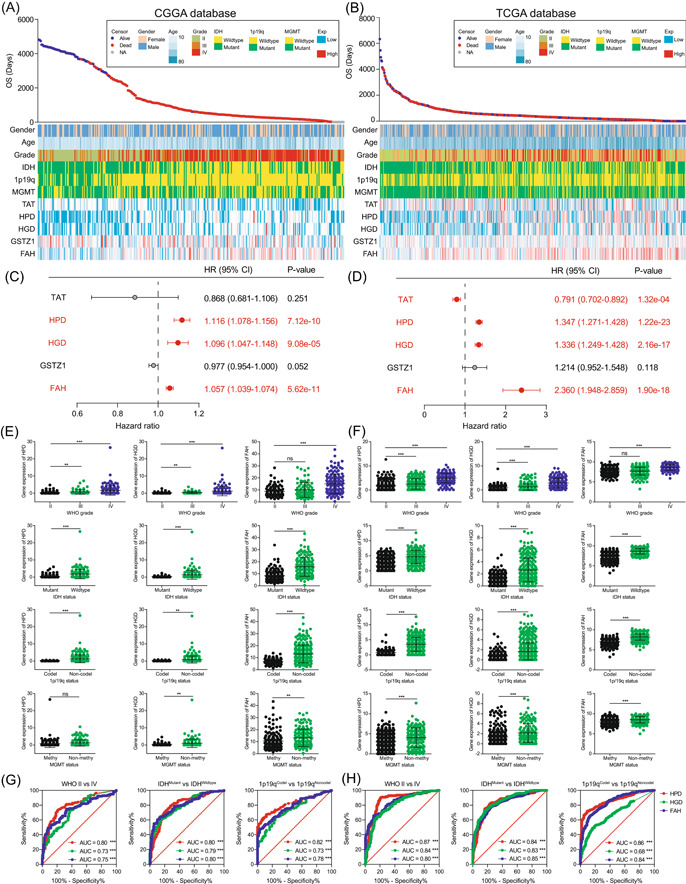
Tyrosine metabolizing enzymes are associated with malignant phenotype and prognosis in glioma patients. The landscape of tyrosine metabolizing enzyme‐related clinicopathological features of glioma in the Chinese Glioma Genome Atlas (CGGA) database (A) and The Cancer Genome Atlas (TCGA) database (B). Univariate Cox's regression analysis for identified prognostic factors (tyrosine aminotransferase [TAT], 4‐hydroxyphenylpyruvate dioxygenase [HPD], homogentisate 1,2‐dioxygenase [HGD], glutathione *S*‐transferase zeta 1 [GSTZ1], and fumarylacetoacetate hydrolase [FAH]) in glioma patients of CGGA database (C) and TCGA database (D). Expression of tyrosine metabolizing enzymes (HPD, HGD, and FAH) in the CGGA (E) database and TCGA (F) database according to World Health Organization (WHO) grade, isocitrate dehydrogenase (IDH) status, 1p/19q status, and O^6^‐methylguanine‐DNA methyltransferase (MGMT) status. The receiver‐operating characteristic (ROC) curve analysis indicated that the tyrosine metabolizing enzymes (HPD, HGD, and FAH) could efficiently distinguish different clinicopathological characteristics (WHO grade, IDH status, and 1p19q status) in glioma patients of the CGGA database (G) and the TCGA database (H). AUC, area under the curve; CI, confidence interval; HR, hazard ratio; OS, overall survival. ***p < 0.001; **p < 0.01; ns indicates *p* > 0.05. [Color figure can be viewed at wileyonlinelibrary.com]

To explore the role of tyrosine metabolism in glioma patients, we ranked the patients according to survival time and survival status and looked for potential associations between the expression levels of tyrosine metabolizing enzymes and the clinical characteristics of patients. The results of the analysis showed that patients (training set) with differential expression of tyrosine metabolizing enzymes exhibited different survival prognoses and clinical characteristics (Figure 1A). The same results were observed in another cohort (validation set) of glioma patients from the TCGA database (Figure 1B). To clarify the correlation of specific tyrosine metabolizing enzymes with the survival prognosis of glioma patients, we used univariate Cox's regression analysis to explore the influence of the expression levels of five tyrosine metabolizing enzymes on the survival prognosis of patients. Among the patients in the CGGA database, we found that HPD, HGD, and FAH significantly affected the patient's survival status and were unfavorable prognostic factors (Figure 1C). On the other hand, tyrosine aminotransferase (TAT), HPD, HGD, and FAH were identified as unfavorable prognostic factors in glioma patients in the validation set (Figure 1D). Taking the above results into consideration, HPD, HGD, and FAH were identified as potential negative prognostic factors for glioma.

To investigate the relationship between the tyrosine metabolizing enzymes and the clinical characteristics of glioma patients, a comparative analysis was conducted with different groups of these samples. In the CGGA database, the expressions of HPD, HGD, and FAH were significantly elevated in high‐grade glioma and IDH wild‐type glioma (Figure 1E). Furthermore, patients without 1p/19q codeletion also exhibited high expression of HPD, HGD, and FAH. The expression of HPD, HGD, and FAH had the same trend in samples without MGMT promoter methylation, although this difference was not statistically significant (Figure 1E). The above results were validated in the glioma patients from the TCGA database (Figure 1F). In addition, TAT and glutathione *S*‐transferase zeta 1 (GSTZ1) also slightly promotes the malignant phenotype of gliomas (Supporting Information: Figure 1). Taken together, these results suggest that the tyrosine metabolizing enzymes HPD, HGD, and FAH promote the malignant phenotype of glioma, which in turn exacerbates the poor prognosis of patients.

Next, we used receiver‐operating characteristic (ROC) curves to study the discriminative ability of tyrosine metabolizing enzymes for different clinical features. As shown in Figure 1G,H, HPD, HGD, and FAH were able to clearly distinguish glioma patients with different characteristics (including WHO grade, IDH mutation status, and 1p/19q codeletion status) in two patient cohorts, suggesting that the expression level of tyrosine metabolizing enzymes could be used as a criterion to classify the malignant degree of glioma. These results indicate that the expression of tyrosine metabolizing enzymes is not only closely related to the clinical characteristics and survival prognosis of glioma patients but also a promising biomarker for the malignant degree of glioma.

### 3.2 Highly expressed tyrosine metabolizing enzymes mediate adaptive immune responses

**Figure 2:**
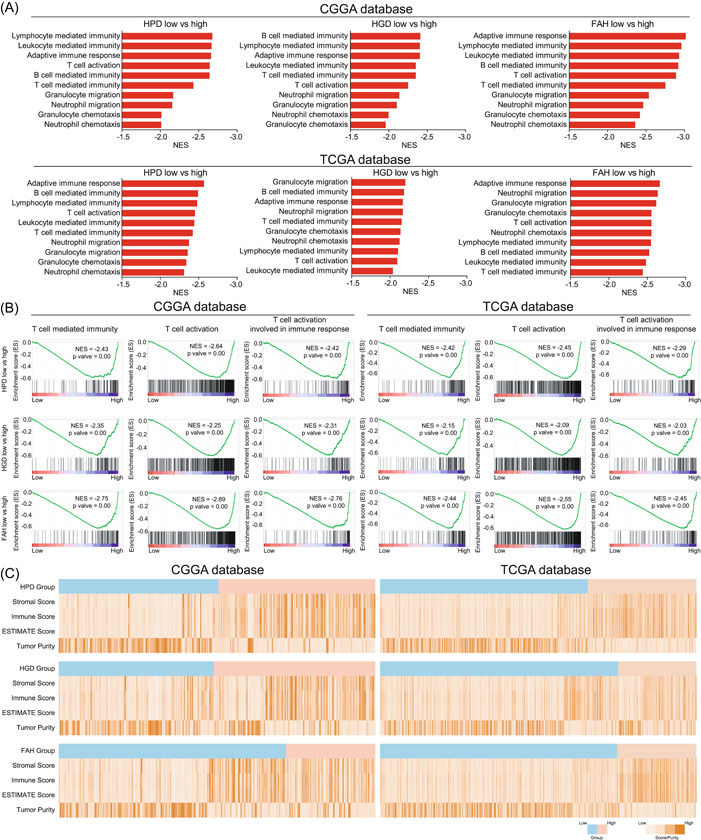
Tyrosine metabolizing enzymes regulate adaptive immunity and immune infiltration in glioma patients. (A) The signaling pathways involved in 4‐hydroxyphenylpyruvate dioxygenase (HPD), homogentisate 1,2‐dioxygenase (HGD), and fumarylacetoacetate hydrolase (FAH) were determined based on normalized enrichment score (NES) values. (B) Gene Ontology (GO) pathway enrichment analyses in the high HPD, HGD, and FAH expression group of glioma patients from the Chinese Glioma Genome Atlas (CGGA) database and The Cancer Genome Atlas (TCGA) database were analyzed by gene set enrichment analysis (GSEA). (C) Heat maps showed immune infiltration scores between low‐ and high‐expression groups of glioma patients from the CCGA database and TCGA database. [Color figure can be viewed at wileyonlinelibrary.com]

To clarify the specific mechanism of tyrosine metabolizing enzymes regulating the malignant phenotype of glioma, we first calculated the optimal “cutpoint” using the R package algorithm and divided glioma patients into two groups according to the expression levels of HPD, HGD, and FAH (Supporting Information: Figure 2A,B). Furthermore, we used the patient's survival prognosis to correct the accuracy and reliability of the grouping. The results showed that the high‐expression group of HPD, HGD, and FAH significantly shortened the survival time of patients in our group (Supporting Information: Figure 2C,D). Among the above high‐ and low‐expression groups, we used pathway enrichment analysis to identify the signaling pathways involved in HPD, HGD, and FAH in glioma. Surprisingly, numerous immune‐related signaling pathways, including adaptive immune response, leukocyte‐mediated immunity, lymphocyte‐mediated immunity, T‐cell‐mediated immunity, and B‐cell‐mediated immunity were identified (Figure 2A). In addition, we also found that T‐cell‐mediated immunity, T‐cell activation, and T‐cell activation involved in immune response were significantly enriched in HPD, HGD, and FAH high‐expression groups (Figure 2B), suggesting that the highly expressed tyrosine metabolizing enzymes are involved in the T‐cell activation process, thereby affecting the immune response. These findings suggested that HPD, HGD, and FAH on glioma cells probably play an essential role in immune response and are involved in T‐cell activation.

### 3.3 PD‐L1 expression increases with increased tyrosine metabolism

**Figure 3:**
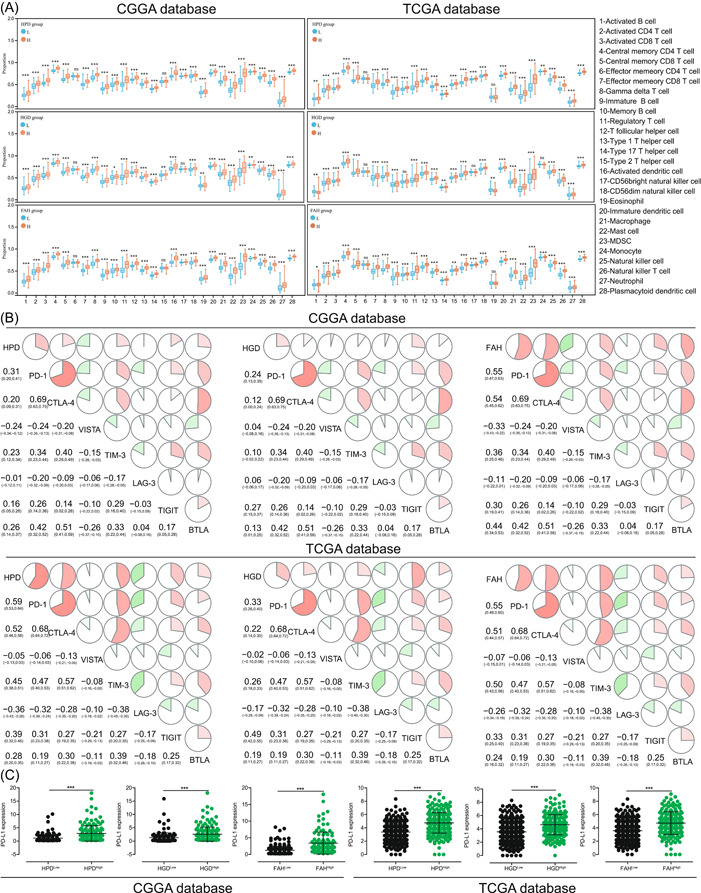
Elevated expression of tyrosine metabolizing enzymes was accompanied by increased expression of programmed death‐ligand 1 (PD‐L1). (A) The difference of multiple immune infiltrating cells between low expression and high expression groups of glioma patients from the Chinese Glioma Genome Atlas (CGGA) database and The Cancer Genome Atlas (TCGA) database. (B) The correlation matrix of tyrosine metabolizing enzymes and immune checkpoint‐related genes. The bottom left showed the correlation coefficient. The correlation coefficients were demonstrated as the proportion of the pie charts. The red parts represented a positive correlation, and the green parts represented a negative correlation. The correlation was tested by Pearson's correlation analysis in the CGGA and TCGA databases. (C) The PD‐L1 expression in tyrosine metabolizing enzyme high‐ and low‐expression groups. FAH, fumarylacetoacetate hydrolase; HGD, homogentisate 1,2‐dioxygenase; HPD, 4‐hydroxyphenylpyruvate dioxygenase. ***p < 0.001; **p < 0.01; *p < 0.05; ns indicates *p* > 0.05. [Color figure can be viewed at wileyonlinelibrary.com]

The TME is composed of tumor cells, immune cells, and surrounding stromal cells that regulate tumor growth and immune responses. We evaluated the effects of HPD, HGD, and FAH on the glioma microenvironment based on the ESTIMATE algorithm. The results showed that both stromal and immune scores were relatively high in the high‐expression group compared with the low‐expression group (Figure 2C). In contrast, the results for tumor purity were relatively high in the low‐expression group. These results suggest that tyrosine metabolizing enzymes promote immune infiltration. Studies have shown that glioma patients with a high degree of immune infiltration have a worse prognosis.^27^ To elucidate the specific mechanism by which tyrosine metabolizing enzymes regulate the immune process of glioma, we used the R package “GSVA” algorithm designed to explore immunity between the low‐ and high‐expression groups. The results showed that most immune cells including activated CD4 T cells, activated CD8 T cells, central memory CD4 T cells, central memory CD8 T cells, gamma delta T cells, and regulatory T cells were all elevated in the high‐expression group (Figure 3A).

In the adaptive immunity process, T cells are the backbone of tumor cell killing. However, activation of T cells requires not only antigen presentation but also the involvement of immune checkpoints. To this end, we analyzed the expression correlation of tyrosine metabolizing enzymes and known immune checkpoints. The results showed that tyrosine‐metabolizing enzymes were positively correlated with most immune checkpoints (Figure 3B). Notably, PD‐1 was the only immune checkpoint significantly associated with the three tyrosine‐metabolizing enzymes. PD‐L1 expressed on the surface of tumor cells could bind to PD1 on the surface of T cells and inhibit activation in T cells.^28^ Interestingly, we found that high expression of HPD, HGD, and FAH upregulated the expression of PD‐L1 (Figure 3C), which indicated that the high expression of tyrosine‐metabolizing enzymes would inhibit the activation of T cells. In all, the highly expressed HPD, HGD, and FAH in glioma modulate the TME, alter the abundance of immune cells, and promote immune evasion.

### 3.4 The metabolite fumarate mediates the malignant phenotype of glioma

**Figure 4:**
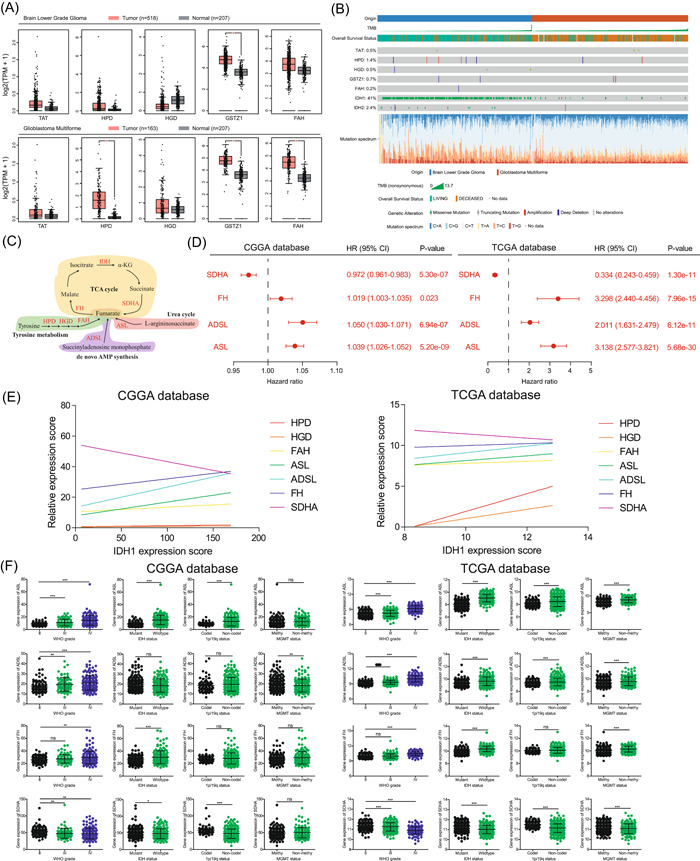
Role of tyrosine metabolizing enzymes depends on tyrosine metabolizing enzyme activity in glioma patients. (A) The expression levels of tyrosine metabolizing enzymes in low‐grade glioma and glioblastoma. (B) The mutation frequencies of tyrosine metabolizing enzymes and isocitrate dehydrogenase (IDH) in low‐grade glioma and glioblastoma. (C) The schematic diagram of fumarate‐related metabolic pathways. (D) Univariate Cox's regression analysis for identified prognostic factors (argininosuccinate lyase [ASL], adenylosuccinate lyase [ADSL], fumarate hydratase [FH], and succinate dehydrogenase complex flavoprotein subunit A [SDHA] in glioma patients of the Chinese Glioma Genome Atlas (CGGA) database and The Cancer Genome Atlas (TCGA) database. (E) The expression correlation of identified metabolic enzymes and IDH1 in glioma patients of CGGA database and TCGA database. (F) Fumarate‐related metabolizing enzymes (ASL, ADSL, FH, and SDHA) expression in the CGGA database and TCGA database according to World Health Organization (WHO) grade, IDH status, 1p/19q status, and O^6^‐methylguanine‐DNA methyltransferase (MGMT) status. CI, confidence interval; HR, hazard ratio. ***p < 0.001; **p < 0.01; *p < 0.05; ns indicates *p* > 0.05. [Color figure can be viewed at wileyonlinelibrary.com]

To further explore the molecular mechanism by which tyrosine metabolism regulates the malignant phenotype and immune response of gliomas, we divided gliomas into low‐grade gliomas (LGGs) and GBMs to explore the expression differences of tyrosine metabolism enzymes. The results showed that the elevated expression of HPD, HGD, and FAH occurred only in high‐grade GBMs(Figure 4A). In addition, we also analyzed the mutation frequency of tyrosine metabolizing enzymes in low‐grade glioma and GBM and found that the mutation frequency of tyrosine metabolizing enzymes was much lower than that of IDH (Figure 4B). The above analysis results not only verify the relationship between tyrosine metabolism and tumor malignancy but also imply that tyrosine metabolism is activated in GBMs.

The mutation status of IDH is an important criterion for glioma classification. However, we found that the highly expressed tyrosine metabolizing enzyme in GBM was accompanied by the wild type of IDH. Therefore, we speculate that tyrosine metabolism and IDH are potentially related. To this end, we sorted out the tyrosine metabolic pathway and IDH‐involved tricarboxylic acid (TCA) cycle and found that the metabolite fumarate may be a key factor (Figure 4C). Subsequently, we also sorted out the fumarate‐related metabolic pathways and related metabolic enzymes including argininosuccinate lyase (ASL), adenylosuccinate lyase (ADSL), fumarate hydratase (FH), and succinate dehydrogenase complex flavoprotein subunit A (SDHA). When we used univariate Cox's regression analysis, we found that ASL, ADSL, and FH were unfavorable factors for glioma patients, while SDHA was a favorable prognostic factor for patients (Figure 4D). In addition, expression correlation analysis showed that the expressions of SDHA and IDH were negatively correlated in the CGGA and TCGA databases, and the rest of the metabolic enzymes we identified were positively correlated with IDH expression (Figure 4E). Besides, we also found that ASL, ADSL, and FH promote the malignant phenotype in glioma patients, while SDHA inhibits malignant progression (Figure 4F). Taken together, these results suggest that fumarate mediates the malignant clinical features and survival prognosis of glioma patients.

### 3.5 Fumarate‐coupled TCA cycle metabolic remodeling regulates PD‐L1 expression

**Figure 5:**
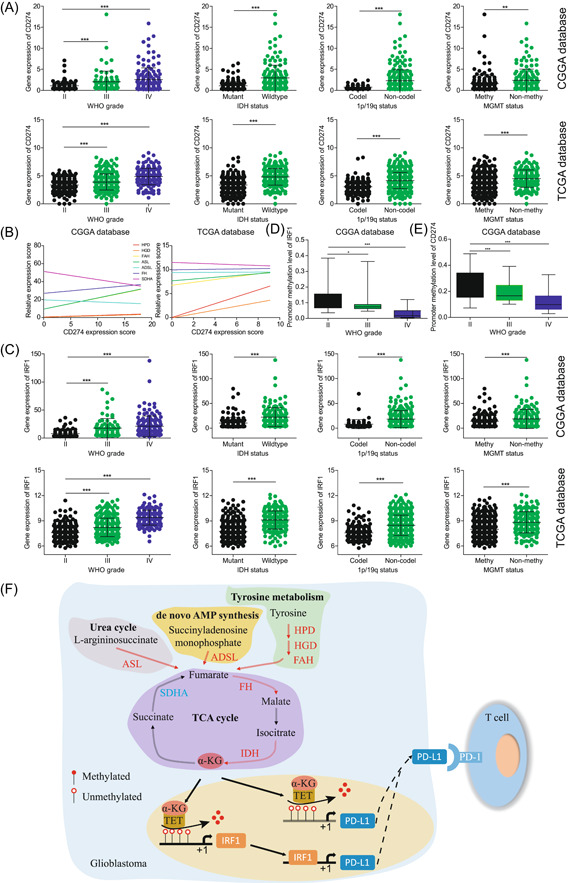
The conversion of high concentrations of fumarate to α‐ketoglutarate (α‐KG) promotes the expression of programmed death‐ligand 1 (PD‐L1) in glioma patients. (A, D) The CD274 (PD‐L1 encoding gene) and IRF1 expression in the Chinese Glioma Genome Atlas (CGGA) database and The Cancer Genome Atlas (TCGA) database according to World Health Organization (WHO) grade, isocitrate dehydrogenase (IDH) status, 1p/19q status and O^6^‐methylguanine‐DNA methyltransferase (MGMT) status. (B) The expression correlation of identified metabolic enzymes and CD274 in glioma patients of the CGGA database and TCGA database. (C, E) The IRF1 and CD274 gene promoter methylation levels were analyzed with different WHO grades of glioma patients in the CGGA database. ADSL, adenylosuccinate lyase; ASL, argininosuccinate lyase; FAH, fumarylacetoacetate hydrolase; FH, fumarate hydratase; HGD, homogentisate 1,2‐dioxygenase; HPD, 4‐hydroxyphenylpyruvate dioxygenase; SDHA, succinate dehydrogenase complex flavoprotein subunit A; TCA, tricarboxylic acid; TET, tet methylcytosine dioxygenase. ***p < 0.001; **p < 0.01; *p < 0.05. [Color figure can be viewed at wileyonlinelibrary.com]

Tyrosine metabolism is not only associated with malignant phenotypes but also with immune evasion. The expression of PD‐L1 (the protein encoded by CD274) was upregulated with the increase in the degree of malignancy of patients (Figure 5A), suggesting that the malignant phenotype of glioma patients may be caused by the increased expression of PD‐L1, which promotes immune evasion. Consistent with IDH, except for SDHA and CD274, the other metabolic enzymes showed a positive correlation (Figure 5B). These correlation results suggest that we have a link between fumarate, IDH, and CD274.

When we reanalyzed the metabolic flow, we found that SDHA and other metabolic enzymes may be the key to solving the problem. When the expression of all metabolic enzymes increased to promote the production of fumarate, the expression of SDHA was abnormally decreased, and more importantly, wild‐type IDH was converted into α‐ketoglutarate (α‐KG) in the TCA cycle. Therefore, we speculate that α‐KG may bridge the link between fumarate and CD274. Interestingly, α‐KG was reported to bind tet methylcytosine dioxygenase (TET) to regulate the expression of PD‐L1. Lv et al.^29^ found that α‐KG activates tet methylcytosine dioxygenase (TET1) and reduces the methylation level of IRF1 promoter to upregulate the expression of the transcription factor IRF1, which in turn promotes the expression of PD‐L1. Unexpectedly, promoter methylation levels of IRF1 decreased with increasing WHO grades (Figure 5C). Moreover, the expression of IRF1 was found to be upregulated with increasing malignancy in the CGGA and TCGA databases (Figure 5D). Unlike this, Verdura et al.^30^ found that α‐KG binds TET, exerts demethylase activity, reduces the methylation degree of PD‐L1 promoter, and regulates the expression of PD‐L1. In both GBM and high‐grade gliomas, the methylation level on the promoter of PD‐L1 was significantly downregulated (Figure 5E), suggesting that PD‐L1 is regulated by promoter methylation modification. Collectively, these results suggest that the tyrosine metabolite fumarate increases conversion to α‐KG through metabolic reprogramming, regulates PD‐L1 expression, and helps glioma cells evade immune surveillance.

## 4 DISCUSSION

Some articles have reported that amino acid metabolism is not only closely related to the survival and prognosis of patients^31^ but also plays an important role in chemotherapy resistance and TME.^32^ Tyrosine metabolism is an important part of amino acid metabolism. In our previous work, it was found that the tyrosine metabolizing enzyme HPD regulates the expression of G6PD, mediates the pentose phosphate pathway, and regulates glucose metabolic flux and DNA synthesis in lung cancer.^33^ Furthermore, HPD is also a prognostic predictor and a potential therapeutic target in breast cancer.^34^ Surprisingly, tyrosine metabolism was significantly downregulated in liver and kidney cancers, which in turn activated the cell cycle and promoted tumor progression.^35^, ^36^ The refractory glioma is largely due to the heterogeneity of the tumor, so identifying the subtype of glioma has guiding significance for its clinical treatment. This study starts from cellular metabolism, looks for the correlation between tyrosine metabolism and clinical manifestations of glioma patients, and further explores the possibility of therapeutic strategies targeting tyrosine metabolism.

The expression of tyrosine metabolizing enzymes has a significant correlation with the malignant phenotype of glioma patients and also has predictive value on the prognosis of patients. These undoubtedly suggest that tyrosine metabolism plays a key role in the progression of glioma. In the in‐depth analysis of tyrosine metabolism regulating the malignant phenotype of glioma, we found that tyrosine metabolism regulates the immune process of glioma, especially adaptive immunity. This is consistent with previous reports that tyrosine metabolism is not only relevant to neurodegenerative diseases (Parkinson's disease)^37^ but also can alleviate inflammatory responses,^38^ all of which suggest that tyrosine metabolism plays an important role in the immune system. Due to the existence of the blood–brain barrier, drug therapy is difficult to achieve a better therapeutic effect for glioma. Therefore, the development of new strategies including immunotherapy is imperative. The involvement of tyrosine metabolism in the immune process of glioma prompted us to further explore its mechanism and provide theoretical help for the subsequent immunotherapy of glioma.

In this study, we intend to establish tyrosine metabolic subtypes to indicate different immune groups, and then provide guidance for clinical treatment of glioma. To this end, we used machine learning methods to optimally divide glioma patients into high‐ and low‐expression groups of tyrosine metabolizing enzymes and corrected the groups through survival prognosis analysis to ensure that the regulation of tyrosine metabolizing enzymes is meaningful for patient prognosis. Immune infiltration was used to evaluate the relative proportions of tumor cells and immune cells in the TME. Generally speaking, the higher the infiltration of immune cells, the lower the purity of tumor cells, and the better the therapeutic effect. However, we found that tyrosine metabolizing enzymes promote the infiltration of immune cells and reduce the purity of tumor cells. Consistent with previous reports, gliomas with a high degree of immune infiltration represented a worse prognosis, increasing the aggressiveness of glioma cells.^27^ In addition, as the expression of tyrosine metabolizing enzymes increased, the proportion of various immune cells also increased, which once again proved the conclusion that tyrosine metabolism promotes immune infiltration. In previously identified adaptive immunity, activation of T cells is a central step in the immune response. In addition to antigen presentation, T cell activation is also inhibited by immune checkpoints. To this end, we analyzed the expression correlation between tyrosine metabolizing enzymes and immune checkpoint‐related genes and found that most immune checkpoints increased with the expression of tyrosine metabolizing enzymes. In glioma cells with high expression of tyrosine, although immune infiltration is increased, the expression of immune checkpoints is increased, resulting in the inhibition of T cell activation and the inability to play a tumor‐killing role.

Although all our results imply a close link between tyrosine‐metabolizing enzymes and tumor progression in glioma patients, we cannot rule out that these metabolic enzymes function independently of metabolic activity. When teasing out the relevant metabolic pathways, we found that the tyrosine metabolites fumarate and IDH are colocated in the TCA cycle. All fumarate‐related metabolic enzymes could significantly affect the malignant phenotype and survival prognosis of glioma patients. More importantly, the abnormally high expression of HPD, HGD, and FAH was always accompanied by wild‐type IDH. These results prompted us to speculate that high concentrations of fumarate are converted to α‐KG in the TCA cycle. In addition to the malignant phenotype, tyrosine metabolism also regulates immune evasion and PD‐1 expression. The expression of PD‐L1 was also upregulated with the degree of malignancy, suggesting that PD‐L1‐promoted immune evasion and malignant phenotype are interrelated. Glioma has been shown to be an immunosuppressive tumor that suppresses the immune system through multiple mechanisms.^39^, ^40^, ^41^, ^42^, ^43^, ^44^ Furthermore, PD‐L1 expression was upregulated in IDH wild‐type glioma, promoting immune evasion, accompanied by increased immune infiltration.^45^ In contrast, PD‐L1 expression was significantly inhibited upon FH deficiency.^46^ Therefore, tyrosine metabolism‐driven fumarate accumulation coupled with the metabolic activity of IDH in the TCA cycle is likely to be an intrinsic mechanism for regulating PD‐L1 expression. The α‐KG could bind to the demethylase TET to promote the expression of transcription factor IRF1 and upregulate the expression of PDL‐1.^29^ On the other hand, it could also directly modify the methylation level of the PD‐L1 promoter and activate gene transcription.^30^ Our results showed that the expression of IRF1 was significantly increased in high‐grade gliomas, and the promoter methylation level of PD‐L1 was also significantly decreased. The metabolic reprogramming of fumarate‐α‐KG driven by tyrosine metabolic enzymes to regulate PD‐L1 expression will provide theoretical support and direction for future glioma immunotherapy.

## 5 CONCLUSIONS

In conclusion, this study explored the association between tyrosine metabolism and the clinical characteristics of glioma patients and found that tyrosine metabolism can not only indicate the malignancy of glioma but also predict the survival prognosis of glioma patients. More importantly, on the one hand, tyrosine metabolism promotes immune infiltration and increases the invasiveness of glioma, and on the other hand, it upregulates the expression of immune checkpoints on the surface of tumor cells, inhibits the activation of T cells, and helps tumor cells to escape immune evasion. By exploring the relationship between metabolic reprogramming and glioma, we want to provide new ideas for the classification of glioma subtypes, provide a theoretical basis for the immunotherapy of glioma, and explore new directions for the precise treatment of glioma.

## AUTHOR CONTRIBUTIONS

Ji‐Yan Wang, Xin‐Tong Dai, and Qing‐Le Gao interpreted the data and arranged the figures. Ji‐Yan Wang and Chang‐Liang Shan wrote and revised the manuscript. Hong‐Kai Chang participated in the design and review of this study. Ji‐Yan Wang, Shuai Zhang, Chang‐Liang Shan, and Tao He were involved in the overall design and supervision of the work. All authors have read and approved the final version of the manuscript.

## CONFLICT OF INTEREST STATEMENT

The authors declare no conflict of interest.

## ETHICS STATEMENT

Not applicable.

## Supporting information

Supporting information.

## Data Availability

The additional data are available in the supporting information and can also be made available by the corresponding author on reasonable request.
