# Supplementary material for: Tyrosine metabolic reprogramming coordinated with the tricarboxylic acid cycle to drive glioma immune evasion by regulating PD‐L1 expression
Source: Ibrain. 2023 May 22;9(2):133–47. doi: 10.1002/ibra.12107 (PMC10529206; doi:10.1002/ibra.12107)
Supplement: Supplementary file 1 — Supporting information. [file IBRA-9-133-s001.docx]

**Tyrosine metabolic reprogramming coordinated with the tricarboxylic acid cycle to drive glioma immune evasion by regulating PD-L1 expression**

Ji-Yan Wang^1^, Xin-Tong Dai^1^, Qing-Le Gao^1^, Hong-Kai Chang^1^, Shuai Zhang^2^, Chang-Liang Shan^1^, Tao He^3^

^1^State Key Laboratory of Medicinal Chemical Biology, College of Pharmacy and Tianjin Key Laboratory of Molecular Drug Research, Nankai University, Tianjin 300350, China

^2^School of Integrative Medicine, Tianjin University of Traditional Chinese Medicine, Tianjin 301617, China

^3^Departments of Pathology, Characteristic Medical Center of The Chinese People’s Armed Police Force, Tianjin, 300162, China

**Correspondence**

Chang-Liang Shan, College of Pharmacy, Nankai University, Tianjin 300350, China

E-mail: changliangshan@nankai.edu.cn

Tao He, Departments of Pathology, Characteristic Medical Center of The Chinese People’s Armed Police Force, Tianjin, 300162, China

E-mail: Hetao_1981@163.com

Ji-Yan Wang, College of Pharmacy, Nankai University, Tianjin 300350, China

E-mail: wangjiyan@nankai.edu.cn

Ji-Yan Wang and Xin-Tong Dai contributed equally to this work.

**Supplemental Figures**


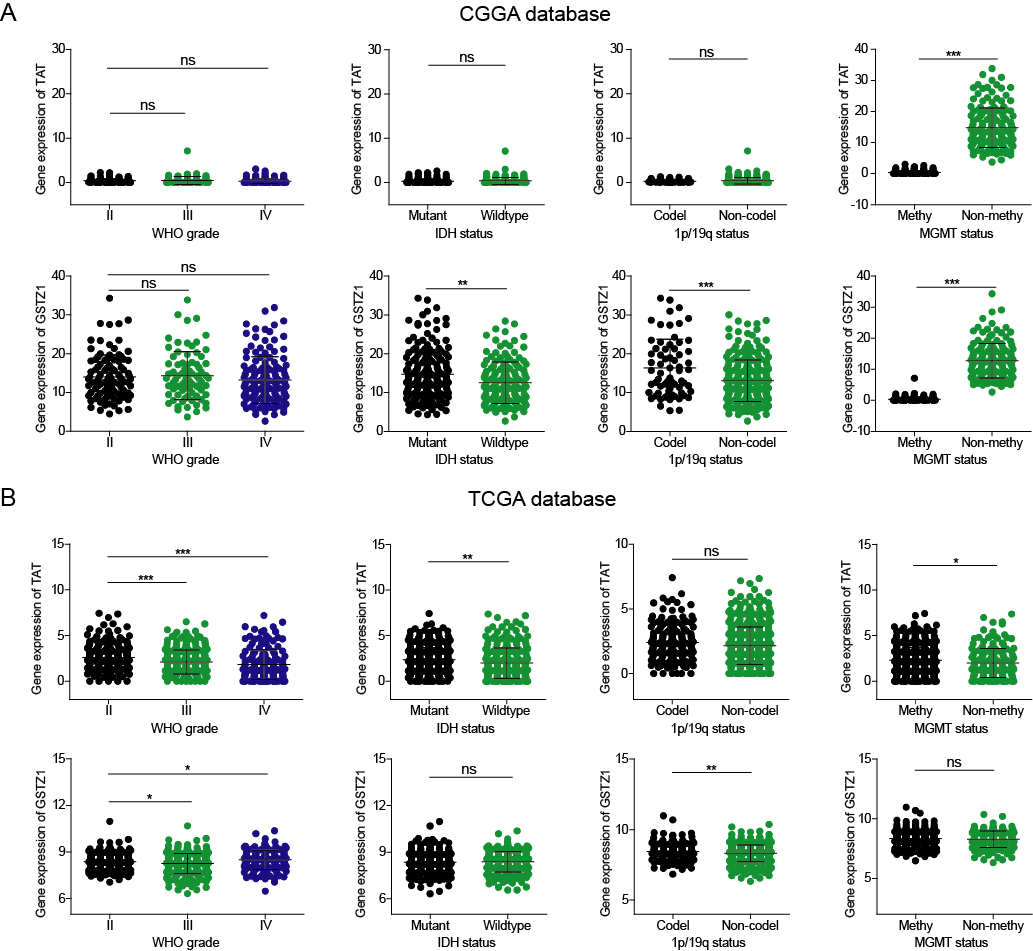


**Supplemental Figure.1** Tyrosine metabolizing enzymes (GSTZ1 and SDHA) expression in CGGA (A) database and TCGA (B) database according to WHO grade, IDH status, 1p/19q status and MGMT status.

**
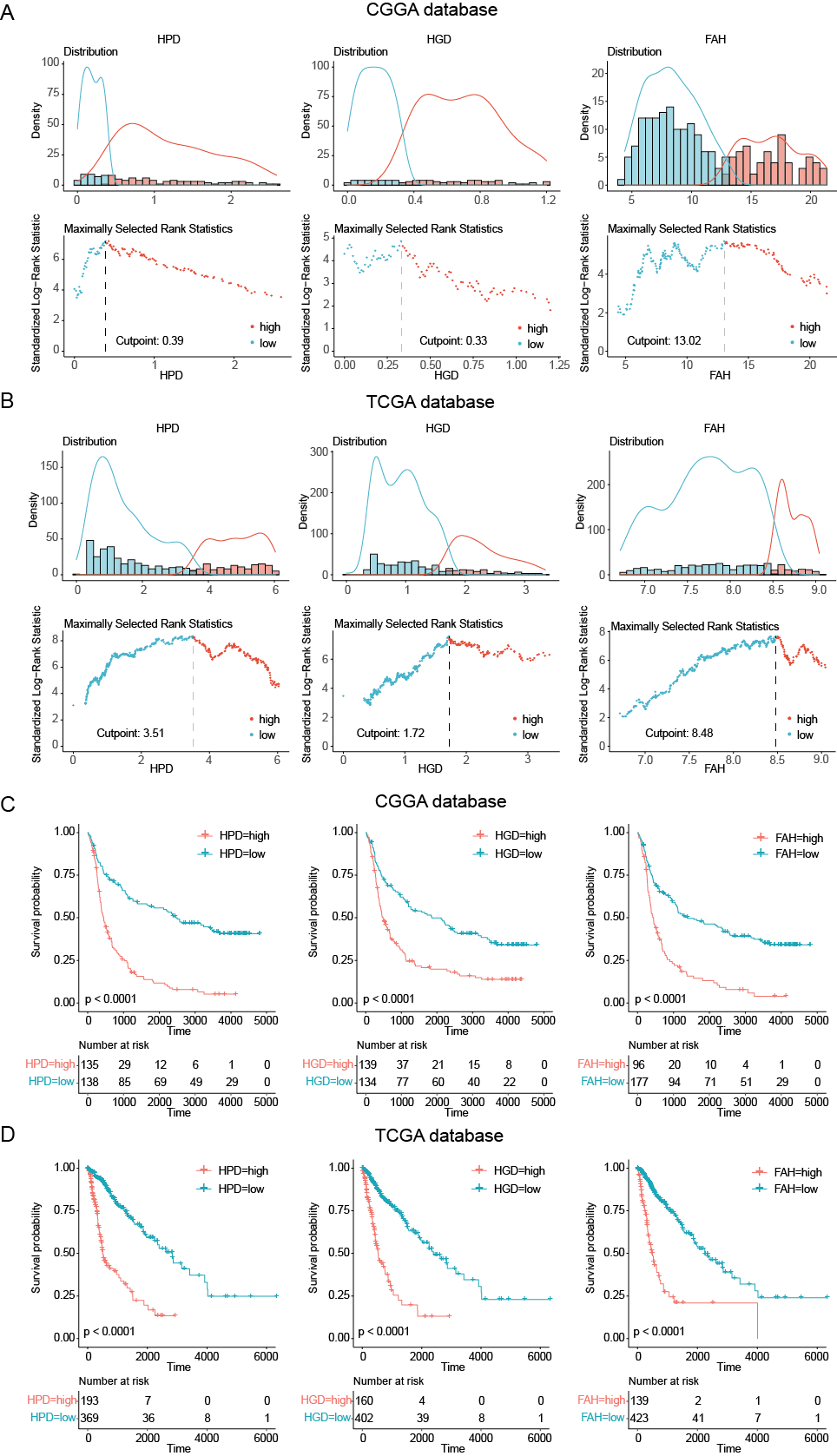
**

**Supplemental Figure.2** The glioma patients were divided into high and low expression groups of specific genes by calculating “cutpoint”. We used the R package "survminer" to calculate cutpoints of tyrosine metabolizing enzymes HPD, HGD and FAH, and divided glioma patients from different databases (CGGA (A) database and TCGA (B) database) into two groups. Survival analysis of glioma patients between low expression and high expression of HPD, HGD and FAH in patients with glioma from CGGA (C) database and TCGA (D) database.
